# Asymptotic BLSCP models in uncertain and asymmetric environments—Take the charging station location problem

**DOI:** 10.1371/journal.pone.0305669

**Published:** 2025-01-24

**Authors:** Muren, Yihao Li, Zhiqiang Xu

**Affiliations:** School of Statistics, Jilin University of Finance and Economics, Changchun, Jilin, China; Cyprus International University Faculty of Engineering: Uluslararasi Kibris Universitesi Muhendislik Fakultesi, TURKEY

## Abstract

The rapid development of the field of vehicles exposes many problems of charging station, the most common is the uncertainty and asymmetry of its task volume, so the flexibility of location and scale design is crucial. This paper proposes the asymptotic BLSCP model, suitable for uncertain and asymmetric environments, which balances facility workload and minimizes cost through the forward or backward asymptotic methods of candidate center locations and flexible allocation of jurisdictions. Additionally, the findings suggest these methods possess considerable potential for application and generalization.

## Introduction

In recent times, governments globally have implemented measures to promote urban transportation system electrification, with a view to enhancing national ecological and energy safety. Notably, Poland aims to diminish environmental pollution by encouraging consumer preferences for electric vehicles and eco-friendly transportation [1]. Electric vehicles have the potential to be highly efficient transportation with almost "zero pollution," but they also require a significant amount of infrastructure and supportive policies. Therefore, it is crucial to continually invest in supporting charging infrastructure [2]. Due to insufficient electric vehicle charging infrastructure across various countries, there is increasing resistance to the penetration of electric vehicles despite their rapid ownership growth [3,4]. As a result, the methodical and gradual construction of electric vehicle charging networks has a significant impact on advancing the adoption of electric vehicles.

There are several practical reasons why the current electric vehicle charging network cannot meet the existing needs. First, high-power charging infrastructure is inadequate and lagging. Naas Technology (NASDAQ: NAAS, www.enaas.com) data shows that the ratio of electric vehicles to charging stations in China has reached 1:1, but the number of high-power charging stations remains inadequate. The selection rate for high-power charging stations of 120kW and above with a small market share is as high as 72%, while the user selection rate for charging stations of less than 30kW with a wide popularity is just 2%. Secondly, there is uncertainty and asymmetry in the demand for electric vehicles. The demand for electric vehicles is subject to several uncertainties, including charging time and charging location. As a result, these factors can leave customers with unsatisfied charging needs. Thirdly, there exists a challenge in matching the supply and demand of charging stations. Presently, Naas Technology data shows that 35.2% of individuals are unsatisfied with charging their vehicles in public areas within urban regions. This is due to unsuitable charging station locations and a lack of management. The fundamental problem is the mismatch between the location of charging stations and the demand for charging, which leads to an impossible supply-demand balance between charging stations and electric vehicles. It is urgent to solve the problem of solving the charging demand of electric vehicles with huge ownership in the process of updating and iterating charging technology. As a result, it is imperative to create asymptotic models based on different perspectives for different stages of development. To solve the problem at different stages of technology development, this paper establishes forward and backward asymptotic methods based on the current optimal and global optimal perspectives.

Determining the number and locations of mixed type stations is a classic facility location problem, which has attracted the attention of researchers for a long time. The Location Set Covering Problem (LSCP) has emerged as an important model that attempting to cover all demand nodes within a given coverage distance using the smallest possible number of facilities [5]. Therefore, research has focused on determining how to allocate a fixed number of facilities to cover the maximum number of demand nodes or workloads. This gave rise to the Maximal Covering Location Problem (MCLP), which was originally introduced by Church and ReVelle [6]. However, many real-world applications, such as emergency services [7,8], energy supplementation [9], and interurban transportation [10], have a common feature in that no demand node within the given area can be ignored for any reason. Obviously, only the LSCP model can effectively solve the full coverage problem. Moreover, from a practical point of view, while it is important to pay attention to the coverage amount and coverage efficiency, it is also important to consider the service efficiency. For example, when constructing a mixed-type station, it is necessary to consider the location cost and time cost incurred by consumers. This paper introduces a lower bound constraint into the LSCP model, herein referred to as the Location Set Covering Problem with Lower Bound (LLSCP). Additionally, this study incorporates both upper and lower bound constraints to balance the workload across each service node, resulting in a model we term the Location Set Covering Problem with Balanced Workload (BLSCP). The lower bound allows for better convergence to the optimal solution compared to just an upper bound. This paper will demonstrate the superiority of these two models using a charging station as a case study. Balanced service is important for many real-world services, ensuring that resources are neither overused nor too idle.

The paper is organized as follows. The notations and basic assumptions are presented in Section 3. The relevant mathematical models are presented in Section 4. The relevant case study and model solving is performed in Section 5. And the relevant conclusions are drawn in Section 6.

## Literature review

In recent years, many researchers have studied the location of charging stations in depth. They mainly focus on the three aspects of supply, demand, and how to achieve the matching of supply and demand. However, how to realize the asymptotic balance of supply and demand in the dynamic development process is hardly mentioned. The research results on supply, demand and matching of supply and demand of charging stations considered by related scholars are shown in Table 1.

**Table 1:** Summary of research results on charging station supply, demand uncertainty.

Author	Uncertainty of demand	Uncertainty of supply	Matching supply and demand
**Liu et al.** [11]			√
**Ran et al.** [12]	√	√	√
**Zeng et al.** [13]	√		√
**Gao et al.** [14]	√		
**Sun et al.** [15]	√		
**Xiao et al.** [16]	√		√
**An** [17]	√	√	
**Uslu and Kaya** [18]	√		

As can be seen in Table 1, first, the problem of demand uncertainty has also been the focus of scholars’ research. Ran et al. [12] proposed a stochastic robust programming method to deal with demand uncertainty from the data-driven perspective. Zeng et al. [13] first used the regret-matching (RM) technique to describe the decision-dependent uncertainties of PEV demand. Uslu and Kaya [18] also considered the driving route of electric buses with limited range, the demand between origin and destination, and the demand rate and capacity of charging stations to obtain the optimal location and capacity. Second, the problem of supply uncertainty has received attention from scholars. Khalifa et al. [19] investigated a transportation issue where costs are denoted by octagonal fuzzy numbers (Oct-FN), and the demands are randomly distributed. An [17] chose the stochastic planning method to deal with the uncertainty of charging demand. They consider that public transportation agencies are often more concerned with the expected cost of locating a charging station than the worst-case maximum cost. Khalifa et al. [20] addressed a fractional transportation problem characterized by discounted costs in a neutrosophic environment, with uncertainties in supply, demand, and transportation costs. Utilizing the KKM approach, Khalifa et al. [21] initially transformed the neutrosophic problem into an equivalent deterministic problem. In addition, many other researchers have used geographic information to analyze each candidate node and select the best charging station location based on demand information. Finally, the problem of how to achieve a match between supply and demand is also the main research direction of various scholars. Liu et al. [11] solved the problem of charging station location and charger configuration under the influence of power matching. Ran et al. [12] considered the problem of mismatch due to the price of external power supplies and power loads. Xiao et al. [16] considered the problem of matching the different stages and determined the optimal location for each stage accordingly.

The above analysis shows that scholars have studied the uncertainty of supply and demand in depth. However, the lack of a suitable model to solve the problem of supply and demand balance has been relatively little researched. In fact, this is the key to solving the problem of wasted resources and idle facilities. The demand for charging is likely to change due to factors such as government infrastructure development and increased vehicle usage. It may also lead to changes in the selection of charging station locations. It is the key problem this paper needs to solve that how to capture the dynamically changing charging information in a timely manner, and realize efficient charging station supply and demand matching by considering the global and balance of the charging station location problem. The asymptotic models established in this paper focus on solving the dynamic supply-demand balance problem to avoid wasting resources and further promote the use and popularization of electric vehicles. During the research of charging station location problem, the theory of location problem has also experienced a long development process. Many scholars have developed various mathematical models and algorithms based on different perspectives to solve the location problem under different conditions. Table 2 further summarizes the results of the location models, solution methods and operation scales applied by various scholars.

**Table 2:** Summary of typical models, solution methods, and operational scales.

Author	Model	Solution method	Operational scale
**Liu et al.** [11]	Mixed integer nolinear program	Surrogate-based optimization method & Gurobi	5×15
**Ran et al.** [12]	Two-stage SEV-CS planning model	Monte Carlo simulation &Gurobi	9×12
**Zeng et al.** [13]	Two-stage stochastic programming with decision-dependent uncertainties	Genetic algorithm	5×12
**Gao et al.** [14]	Bilevel mode	Heuristic algorithm	4×13
**Sun et al.** [15]	Mixed-integer bi-level program	Active-set algorithm	26×49
**Xiao et al.** [16]	Queueing model	Genetic algorithm	4×20
**An** [17]	Stochastic integer program	Customised lagrangian relaxation approach	90×119
**Uslu and Kaya** [18]	Mixed integer-linear mathematical model	General Algebraic Modeling System optimization software program using Cplex	6×136

From Table 2 above, it can see that the models proposed by researchers are mainly based on nonlinear mixed integer programming. The solution algorithms are mainly a combination of exact and heuristic algorithms. Among them, the solution scale of the nonlinear mixed integer programming model is small, which is not suitable for the application of large-scale problems. Although the heuristic algorithm significantly increases the solution scale, it cannot be effectively guaranteed in terms of accuracy. Therefore, this paper tries to improve the solution scale and speed of exact algorithms through reasonable variable assumptions to realize the goal of efficient dissemination and application.

By summarizing the above literature, this paper finds that the current research on location problem mainly focuses on three aspects, namely, uncertainty of supply, uncertainty of demand, and matching supply and demand problem. More research has been done on the uncertainty of supply and demand, while less research has been done on the matching supply and demand problem and the dynamic balance problem. However, the challenge of dynamic balance holds significant importance in both resource conservation and the advancement of sustainable travel methods. It will be a top priority for future research. Therefore, this paper considers the balance problem based on the uncertainty and uses the backward algorithm to obtain the optimal solution under different supply and demand environments. This method has a wide range of applications. In terms of the types of models established by scholars, they are mainly nonlinear programming models. These algorithms are slower and smaller in scale compared to the linear programming models. Among the solution methods, most of them are heuristic algorithms, which give approximate results and may be limited in practical application. Therefore, it is urgently needed to propose an accurate algorithm based on linear programming.

Determining the number and location of electric vehicle charging stations is typically a facility location problem. One of the more widely used models is the Location Set Covering Problem (LSCP). And the model has come a long way in its appearance and development. After Toregas et al. [5] proposed the LSCP model to figure out emergency facilities’ location choice, many scholars took it as an effective way to solve practical problems. The more frequency uses of the model also makes the basic theory constantly improved. Just two years later, Walker [22] established set-covering problem model to settle the distribution of fire companies in New York. His work optimizes the number of tower ladders within the condition that rescue more people. After that, the model became more and more complex, it’s scale and scope of application were also expanding. Daskin [23] set the objective of maximizing the expected value of demand coverage in facilities covering location problem (MEXCLP) and use a *p* probability to describe the facilities busyness. Today it can see the application of location theory in many aspects of life, such as bike-sharing and bloodmobile [24].

In the process of studying practical problems, actual conditions are random and variable. Wherefore some scholars realize that such randomness and variability should also be considered when formulating the LSCP model. Berman et al. [25] concentrate on gradual covering location problem with random demands. Zhang et al. [26] took uncertainty theory into emergency service stations location problem to achieve rapid transportation of materials after the disaster. He wrote the delivery time as fuzzy variable because of its uncertainty. Practical problems with random factors have always been research hotspots, and probability theory is a useful tool to deal with random factors, especially fuzzy set theory and uncertainty theory. Fuzzy set theory was proposed to depict the blur of things [27] and uncertainty theory are more used to deal with belief degrees [28]. For uncertain variables, the variables can be transformed into fuzzy sets by describing the appropriate membership functions. The combination of LSCP model and probability theory makes the facilities theory more abundant. Darzentas [29] introduced set covering in the domain of fuzzy sets and developed a fuzzy model for the facility location problem based on fuzzy set partitioning.

The initial LSCP model is not complicated in form and scale, it is feasible to find optimal solutions. But as the theory matures, it has evolved into an NP-hard problem, which is difficult to be solved with an exact algorithm. In recent years, more and more scholars have devoted themselves to the heuristic algorithm for LSCP models. Meng and Shia [30] formulated a new location set covering problem model with stochastic critical distances and use two-path search algorithm solve the problem. Akl et al. [31] applied location set covering model to the allocation of base stations in wireless networks, and proposed 1/2 approximation algorithm. Berman et al. [32] presented theoretical foundations of joint coverage relationship and compared the effects of different algorithms. Owais et al. [33] established genetic algorithm with randomized priority search to implement for the location set covering problem, with a case contain 84 vertices and 143 edges. Vasko et al. [34] explore greedy-like heuristic in weighted set covering problem. To solve Wireless Sensor Networks’ set k-cover problem, Liao and Ting [35] investigated memetic algorithm based on integer coded genetic algorithm and local search.

All those works make LSCP models more than in paper, but due to the constraints of real conditions, the service capabilities of each station do not match the service requirements. Some authors added maximum capacity constraints to the LSCP models, Pirkul and Schilling [36] proposed an upper bound on the total workload that a service center can afford as early as 1991. According to this, Felici et al. [37] used probabilistic method to obtain a-priori upper bound for the Set Covering problem. Umetani et al. [38] studied heuristic algorithm for set covering problem and generalized upper bound (GUB) constraints that arise in many real applications of SCP. Also, in the paper by Akl et al. [31], the location set covering problem is extended by incorporating upper bounds on facilities. However, the only constraint of service capabilities is not comprehensive, which may lead some service nodes have too little demand. For service nodes, low demand means low service efficiency. Adding a lower bound constraint of service capabilities can not only share the high-demand node working pressure, but also avoid the waste of social resources. Salhi and Al-Khedhairi [39] formulated derive potential ‘lower bounds’ according to tight upper bound, but the authors point out that there are some shortcomings in the lower bound proposed in this article that need to be improved.

This paper attempt to set both upper and lower bound in traditional LSCP model and apply it in the establishment of mixed type stations. Miralinaghi et al. [9] studied genetic algorithm in refueling station location problem. There are many similarities between the location of car charging stations and the location of refueling stations. Charging stations can be seen in many refueling stations. Therefore, Miralinaghi et al. [9] thesis suggests that the location problem of charging stations can be effectively addressed through the LSCP model.

This paper proposes BLSCP model under uncertainty after further considering the uncertainty of supply and demand and balance on the top of the traditional LSCP model. The major innovations are summarized as follows:

1. This paper refines the LSCP model by proposing the LSCP model with upper and lower bounds and its certainty model with minimum upper and maximum lower bounds.
2. The balance problem in uncertain environment is fully considered. The charging station location problem in certain and uncertain environments are compared to find the necessity and practical significance of introducing new models.
3. The dynamic change of supply and demand is fully considered. The set of potential candidate nodes is further identified through large-scale demand simulation, which guarantees the rationality of the dynamic location selection results.
4. In this paper, the balanced location problem of the charging station is taken as an example to introduce in depth the value of the generalized application of the BLSCP model in uncertain environment.

## Notation and basic assumption

To address the challenges of optimizing charging station locations amidst uncertainty and asymmetry in demand, this paper develops the asymptotic BLSCP model. In this section all the symbols involved in the models are explained for easier understanding. Mathematical statements of LSCP models rely on the following notation:

*i* index for demand points

*j* index for candidate locations

*I* set of demand points

*J* set of all candidate locations

*T*_*j*_ set of demand point is covered by candidate location *j*

${\bar{w}}_{i}$ coefficient reflecting the desirability of covering demand point *i*

*p*_min_ minimum number of facilities

*ε* upper bound of relaxation balance constrain level

*d*_*ij*_ distance from point *i* to candidate location *j*

*ξ*_*i*_ uncertain variable of demand in point *i*

*C*_*ij*_ unit-weighted transportation cost from point *i* to candidate location *j*

*C*_*j*_ construction cost of the candidate facility *j*

*r* index for service radius

*R* set of all service radius

Decision variables of facilities are:

$$x_{j}=\left\{\begin{array}{l} 1\;\text{if}\;\text{a}\;\text{facility}\;\text{is}\;\text{located}\;\text{at}\;\text{candidate}\;\text{location}\;j\\ 0\;\text{otherwise} \end{array}\right.$$

$$z_{ij}=\left\{\begin{array}{l} 1\;\text{if}\;\text{demand}\;\text{point}\;i\;\text{is}\;\text{served}\;\text{by}\;\text{facility}\;j\\ 0\;\text{otherwise} \end{array}\right.$$

$e_{j}^{+}$ the part of workload that is greater than the ideal workload

$e_{j}^{-}$ the part of workload that is smaller than the ideal workload

Basic assumptions in this paper:

1. the highest service capabilities of facility *j* is defined as *U*_*j*_; while the minimum workload of facility *j* is *L*_*j*_. *U*_*j*_ and *L*_*j*_ are calculated according to actual conditions, *U*_*j*_ is the maximum workload limit and *L*_*j*_ is the minimum standard for facility profitability.
2. all demands are positive real numbers.

### Location set covering problem with balanced workload

Simply speaking, the process of establishing the LSCP model is the process of selecting the least number of points from *J* to cover the whole *I*. In this process each demand node should be serviced by at least one facility in a short period of time under the constraint of realistic.

### BLSCP model within the certain condition

The traditional LSCP model can be used to find the minimal number of facilities. The mathematical expression of the traditional LSCP model is Model 1.

**Model 1**

$$\text{min}\;\sum_{j\in J}x_{j}$$ (1)

$$\begin{array}{ll} s.t. & \;\sum_{j\in T_{I}}x_{j}\geq 1\;\;for\;all\;i\in I\\ & x_{j}\in \{0,1\}\;\;for\;all\;j\in J \end{array}$$ (2)

The objective (1) is to minimize the number of facilities, Constraint (2) ensures each demand node is covered. It can use Model 1 to find out the *p*_min_ in the location problem.

**Fig 1:**
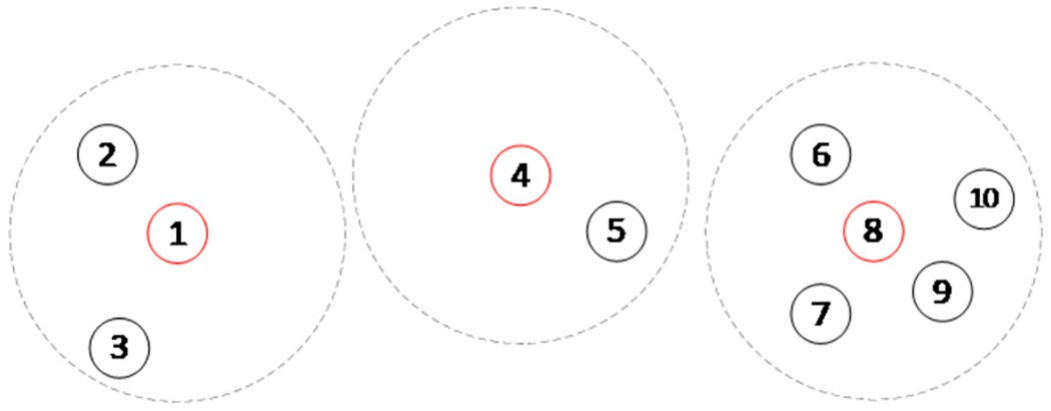
Illustration of LSCP model.

Fig 1 illustrates an example of LSCP with 10 demand nodes, and three facilities can be used, which are selected at 1, 4 and 8, cover all 10 nodes. It is obvious that 8’s coverage requirement is much larger than the 4, that means 8 is far busy than 4. When not considering the balance workload of facility, vertex 8’s requirement is equal to the total demand of vertices in *T*(8) = {6,7,8,9,10}. The waiting time of 8 becomes longer and 4 has more idle resources, it causes the unbalance of workload among the service nodes, which further leads to an increase in the total service time. This paper will establish a new model of LSCP with balanced workload, denoted by BLSCP for short.

After getting *p*_min_ with Model 1, it is necessary to consider the balance of facilities’ workload. The use of a balanced allocation of workloads ensures that the lower bound of the smallest of the service facilities of all the locations is maximized, and the upper bound of the largest of the service facilities of all the locations is minimized. To identify the ideal value of lower bound *L*_*ideal*_ and upper bound *U*_*ideal*_, this paper formulates Model 2 and Model 3.

**Model 2**

$$\text{min}\;U_{ideal}$$ (3)

$$s.t.\;\sum_{j\in J}x_{j}=p_{\text{min}}$$ (4)

$$\sum_{j\in J}z_{ij}=1\;for\;all\;i\in I$$ (5)

$$z_{ij}\leq x_{j}\;for\;all\;i\in T_{j},j\in J$$ (6)

$$z_{ij}=0,i\notin T_{j},j\in J$$ (7)

$$\sum_{T_{j}}{\bar{w}}_{i}z_{ij}\leq U_{ideal}\;for\;all\;\;j\in J$$ (8)

$$x_{j},z_{ij}\in \{0,1\}\;for\;all\;i\in T_{j},j\in J\;U_{ideal}\geq 0$$

The optimal value of Model 2 can be named as *U*_*i0*_, which be used in Model 3 to determine the lower bound of workload. ${\bar{w}}_{i}$ is the expected value of the random variable ${\tilde{w}}_{i}$ at the demand node, i.e., ${\bar{w}}_{i}=E(w_{i})$, where *E*(·) is to solve for the expected value.

**Model 3**

$$\text{min}\;L_{ideal}$$ (9)

$$\begin{array}{ll} s.t. & \text{Constraints}\;\left(4,5,6,7\right)\\ & \sum_{T_{j}}w_{i}z_{ij}\geq L_{ideal}-(1-x_{j})U_{i0}\;for\;all\;\;j\in J\\ & x_{j},z_{ij}\in \{0,1\}\;\;for\;all\;i\in T_{j},j\in J\;L_{ideal}\geq 0 \end{array}$$ (10)

Expression -(1-*x*_*j*_)*U*_*i0*_ in Constraint (9) can make sure all the lower bounds are reasonable even if the candidate locations are not selected.

In this way the ideal workload of each facility can be defined as *w*_*ideal*_ = *αL*_*ideal*_+(1-*α*)*U*_*ideal*_, where the parameter is in the range α∈(0,1). Thus, this paper formulates Model 4 to balance the workload of facility.

**Model 4**

$$\begin{array}{ll} \text{min} & \sum_{j\in J}{\left(\sum_{i\in T_{j}}w_{i}z_{ij}-w_{ideal}\right)}^{2}\\ s.t. & \text{Constraints}\;\left(4,5,6,7\right)\\ & x_{j},z_{ij}\in \{0,1\}\;for\;all\;i\in T_{j},j\in J \end{array}$$ (11)

This part uses *z*_*ij*_ to trace the facility that each demand point belongs to. The optimal value of Model 4 can be named as *W*_*opt*_.

When the demand is known and fixed, Model 4 can not only balance the workload but also give the optimal allocation of demand points. But the objective is nonlinear, and the model is quadratic programming, it should be transformed into linear programming.

**Model 5**

$$\text{min}\;\sum_{j\in J}e_{j}^{+}+e_{j}^{-}$$ (12)

$$\begin{array}{ll} s.t. & \text{Constraints}\;\left(4,5,6,7\right)\\ & \sum_{i\in T_{j}}w_{i}z_{ij}-x_{j}w_{ideal}+e_{j}^{+}-e_{j}^{-}=0\;for\;all\;j\in J\\ & x_{j},z_{ij}\in \{0,1\}\;\;for\;all\;i\in T_{j},j\in J\\ & e_{j}^{+},e_{j}^{-}\geq 0\;\;for\;all\;j\in J \end{array}$$ (13)

In Model 5, the location of facilities and the allocation of workloads may have the duplicate result. Allocating the demand point with proximity principles request loose balance constraint and close weighted distance between demand node and facility, which are showed in Model 6.

**Model 6**

$$\text{min}\;\sum_{j\in J}\sum_{i\in T_{j}}w_{i}d_{ij}z_{ij}$$ (14)

$$\begin{array}{ll} s.t. & \text{Constraints}\;\left(4,5,6,7,13\right)\\ & \sum_{j\in J}e_{j}^{+}+e_{j}^{-}\leq W_{opt}+\varepsilon \\ & x_{j},z_{ij}\in \{0,1\}\;\;for\;all\;i\in T_{j},j\in J\\ & e_{j}^{+},e_{j}^{-}\geq 0\;\;for\;all\;j\in J \end{array}$$ (15)

### BLSCP model within the uncertainty condition

In practical work, restrictions on real conditions may lead to the bound of workload highly unpredictable. To response the uncertainty, fuzzy theory and uncertainty theory are used during the modeling process. According to the knowledge of probability theory, the sample value of the demand of the facility can be observed, and then estimate the uncertainty demand that is close enough to the actual value with Monte Carlo method. For demand points *i*, its uncertain demand *ξ*_*i*_ can be absorbed by the facility. According to LSCP requirements, each demand node should be covered by at least one facility while using the minimum number of facilities. Also, balanced workload requires facilities should have an ideal workload.

There will be some demand points can get served from more than one facility. The process of redistributing the demand for these nodes who have the right to choose facility will again affect the balance of facility workload. If all those nodes do the same choose, the upper bound of facility makes no sense. There will be a situation that one facility has a long waiting time while the other can’t make ends meet. Every time this paper uses experiments to generate a set of uncertainty demand{*ξ*_*i*_ | *i*∈*I*}, from which it can get the corresponding *L*_min_ and *U*_min_. After several trials, a series of *L*_min_ and *U*_min_ can be obtained. Then the bound of workload can defined as *L* = max{*L*_min_} and *L* = min{*L*_max_}. An ideal workload of each facility can be set according to the upper and lower bound, which is defined as *w*_*ideal*_ = *βL*+(1-*β*)*U*, where the parameter is in the range *β*∈(0,1). The closer the workload of each facility is to the ideal workload, the better the balance.

With the ideal workload *w*_*ideal*_ and *p*_min_ calculated by Model 1, the BLSCP model with uncertainty can be formulated as following:

**Model 7**

$$\begin{array}{ll} \text{min} & \sum_{j\in J}{\left(\sum_{i\in T_{j}}\xi_{i}z_{ij}-w_{ideal}\right)}^{2}\\ s.t. & \text{Constraints}\;\left(4,5,6,7\right)\\ & x_{j},z_{ij}\in \{0,1\}\;for\;all\;i\in T_{j},j\in J \end{array}$$ (16)

The linear form of Model 7 is summarized as Model 8.

**Model 8**

$$\begin{array}{ll} \text{min} & \sum_{j\in J}e_{j}^{+}+e_{j}^{-}\\ s.t. & \text{Constraints}\;\left(4,5,6,7\right)\\ & \sum_{i\in T_{j}}\xi_{i}z_{ij}-x_{j}w_{ideal}+e_{j}^{+}-e_{j}^{-}=0\;for\;all\;j\in J\\ & x_{j},z_{ij}\in \{0,1\}\;for\;all\;i\in T_{j},j\in J\\ & e_{j}^{+},e_{j}^{-}\geq 0\;\;for\;all\;j\in J \end{array}$$ (17)

The minimal objective value of Model 7 is defined as *W*_*opt*_. Although Model 7 and Model 8 can balance the workload, customers may not adopt this balanced allocation strategy. Appropriate relaxation of constraints is a feasible optimization solution, which means that the probability of the facility workload being higher than the lower bound and lower than the upper bound is maximized. This paper uses the Monte Carlo method to simulate the probability that the workload of each facility is higher than the ideal lower bound of the workload *LP*_*j*_ and the probability that the workload of each facility is lower than the ideal upper bound of the workload *UP*_*j*_. Therefore, this research can use Model 9 to get the optimal strategy.

**Model 9**

$$\begin{array}{ll} \text{max} & \sum_{j\in J}(LP_{j}+UP_{j})x_{j}\\ & \text{Constraints}\;\left(4,5,6,7,15,17\right)\\ & \sum_{i\in T_{j}}\xi_{i}z_{ij}-x_{j}w_{ideal}+e_{j}^{+}-e_{j}^{-}=0\;for\;all\;j\in J\\ & e_{j}^{+},e_{j}^{-}\geq 0\;\;for\;all\;j\in J \end{array}$$ (18)

### Asymptotic BLSCP model considering location and weighted transportation costs

During the construction process, a dynamic location plan is more in line with the needs of practical management needs. A rationalized dynamic location plan can significantly reduce the cost and avoid the waste of resources. Therefore, based on the existing model, this paper further expands and establishes the asymptotic BLSCP model considering the location cost and scheduling cost.

Asymptotic location models are mainly categorized into forward location methods and backward location methods. The forward location method refers to the construction process in which the number of locations goes from less to more. This construction method requires the timely addition of new charging stations in response to the increase in charging demand. The backward location method is the process of selecting the number of locations from more to less. It requires planning under ideal demand and then building according to the actual demand at the current stage. The specific model of the forward location method is shown below:

**Model 10**

$$\text{min}\;\sum_{j\in J}\sum_{i\in T_{j}}C_{ij}w_{i}d_{ij}z_{ij}+\sum_{j\in {∁}_{J}J_{R_{sort}(r)}}{C^{\prime }}_{j}x_{j}$$ (19)

$$s.t.\;\;\text{Constraints}\;\left(5,6,7,13,15\right)$$

$$x_{j}=1\;for\;all\;j\in J_{R_{sort}(r)}$$ (20)

$$x_{j}\in \{0,1\}\;for\;all\;j\in {∁}_{J}J_{R_{sort}(r)}\;$$ (21)

$$z_{ij}\in \{0,1\}\;for\;all\;i\in T_{j},j\in J$$

$$e_{j}^{+},e_{j}^{-}\geq 0\;\;for\;all\;j\in J$$

The objective (19) is to minimize location and weighted transportation costs. Where *C*_*ij*_ is the unit-weighted transportation cost and *C*^*’*^_*j*_ is the location cost. In general, the closer the location is to the city center, the higher the cost of the location. In addition, *R*_*sort*_ can be obtained by sorting the elements in the radius set *R* from smallest to largest. Where *S* is the number of elements in the *R*_*sort*_, *R*_*sort*_(*r*) is the *r*-th element in the *R*_*sort*_, and *r* is in the range {1,2,…,*S*}. The Constraint (20) is that the facilities from the previous stages must be considered. The Constraint (21) is to select the facility by removing the result of the previous stage in the set *J*. For example, in the beginning, the construction was based on the *R*_*sort*_(*r*+1) service radius, and the number of facilities was small relatively. As the demand increases, the number of facilities under *R*_*sort*_(*r*+1) radius cannot satisfy the current demand obviously. Therefore, new facilities must be added according to the *R*_*sort*_(*r*) service radius. The facilities constructed in the previous phase do not need to be removed. It is only necessary to select locations among the remaining candidate nodes.

**Model 11**

$$\begin{array}{ll} \text{max} & \sum_{j\in J}(LP_{j}+UP_{j})x_{j}-\sum_{j\in {∁}_{J}J_{R_{sort}(r)}}{C^{\prime }}_{j}x_{j}\\ s.t. & \text{Constraints}\;\left(5,6,7,15,17,20,21\right)\\ & z_{ij}\in \{0,1\}\;\;for\;all\;i\in T_{j},j\in J\\ & e_{j}^{+},e_{j}^{-}\geq 0\;\;for\;all\;j\in J \end{array}$$ (22)

The Model 11 is based on an asymptotic location model in the uncertain environment. The objective (22) is to add location costs to objective (18) in model 9. The constraint on the number of locations is not added to Models 10 and 11. This is because the case that the number of locations is determined may lead to worse balance. Appropriate relaxation of the constraints without limiting the number of locations may lead to more reasonable results.

The specific model of backward location method is shown below:

**Model 12**

$$\text{min}\;\sum_{j\in J}\sum_{i\in T_{j}}C_{ij}w_{i}d_{ij}z_{ij}+\sum_{j\in J_{R_{sort}(r)}}{C^{\prime }}_{j}x_{j}$$

$$s.t.\;\text{Constraints}\;\left(5,6,7,13,15\right)$$

$$x_{j}=0\;for\;all\;j\in {∁}_{J}J_{R_{sort}(r)}$$ (23)

$$x_{j}\in \{0,1\}\;for\;all\;j\in J_{R_{sort}(r)}$$ (24)

$$z_{ij}\in \{0,1\}\;\;for\;all\;i\in T_{j},j\in J$$ (25)

$$e_{j}^{+},e_{j}^{-}\geq 0\;\;for\;all\;j\in J$$

The model 11 is based on an asymptotic location model in a certain environment. The Constraint (23) and Constraint (24) are that the current stage of the facility should be based on planning for a future stage. The Constraint (25) is that the facility for the current stage is selected from the facilities selected for the future stage. For example, after initializing the final *R*_*sort*_(1) radius location results, the *R*_*sort*_(2) radius location results must be determined based on the final stage.

Similarly, a backward location selection method for uncertain environments can be established. The specific model is shown below:

**Model 13**

$$\begin{array}{ll} \text{max} & \sum_{j\in J}(LP_{j}+UP_{j})x_{j}-\sum_{j\in J_{R_{sort}(r)}}C_{j}x_{j}\\ s.t. & \text{Constraints}\;\left(5,6,7,15,17,23,24,25\right)\\ & z_{ij}\in \{0,1\}\;for\;all\;i\in T_{j},j\in J\\ & e_{j}^{+},e_{j}^{-}\geq 0\;for\;all\;j\in J \end{array}$$

**Fig 2:**
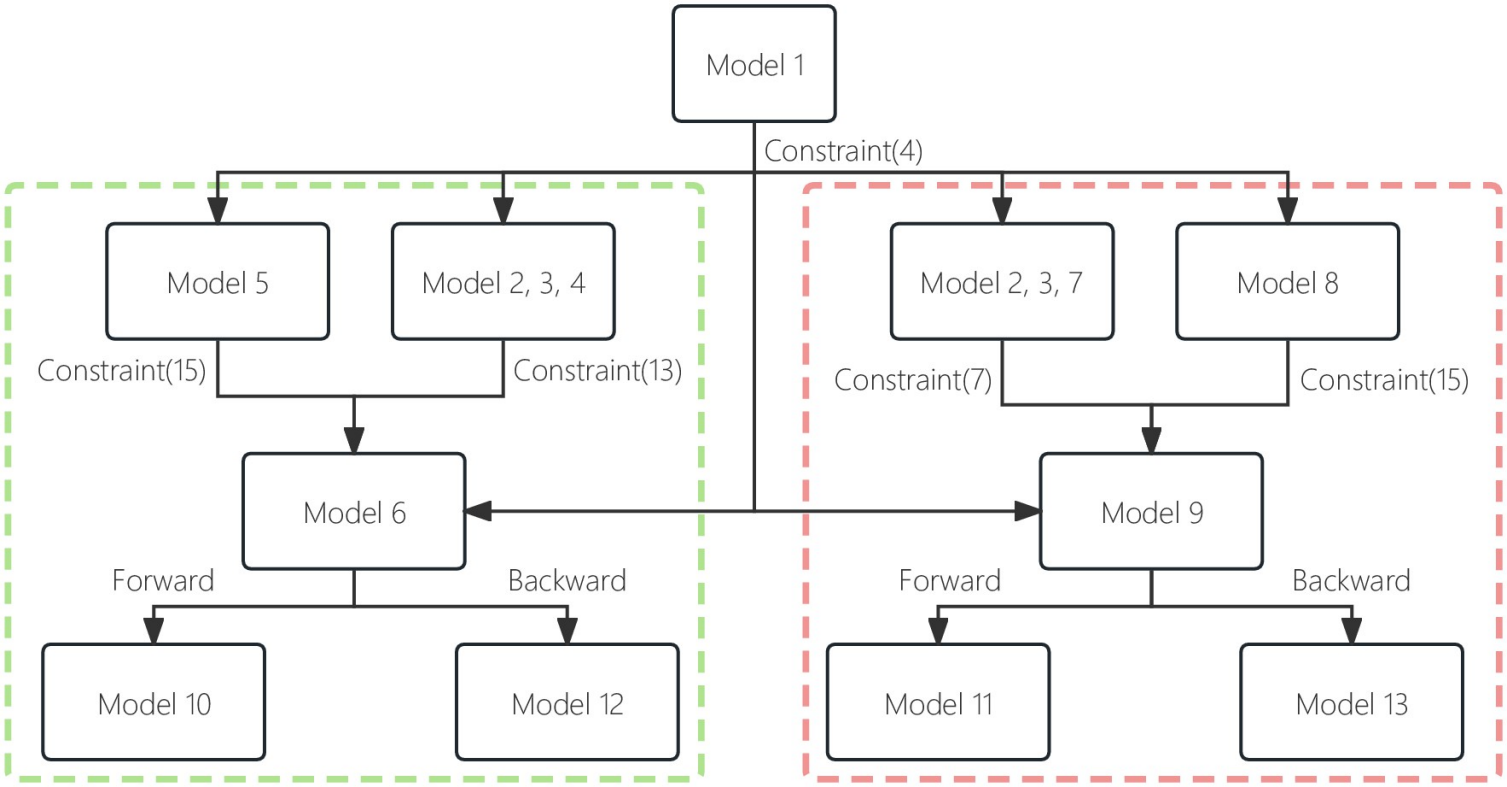
Model relationship in different environments.

To succinctly express the connections between the models in this paper, a relationship diagram was created. The specific results are shown in Fig 2.

In Fig 2, the green and red dashed boxes show the models used in the certain and uncertain environments, respectively. In the certain environment of the forward method, first model 1 provides the parameter *p*_min_ for the Constraint (4) of models 2, 3, 4, and 5. Second, models 2, 3, and 4 are used to provide the parameter *w*_*ideal*_ for the Constraint (13) of model 6. At the same time, model 5 provides parameter *W*_*opt*_ for the Constraint (15) of model 6. Model 6 is the initial model in the certain environment of the forward method. Finally, the location problem can be solved asymptotically using model 10.

## Case study

### Data collection and preparation

This paper takes a city in China as an example to study the distribution of charging stations. An [17] selected 119 candidate locations and applied them to a larger research scope. Therefore, a total of 141 candidate locations were selected and the relevant data collected for the city is shown in Appendix at the end of the paper. In this paper, different location plans can be obtained by using different service radii. The aim is to obtain asymptotic optimization results. When the service radius is small, it indicates a higher demand for charging stations in the area and more electric vehicle usage. In the contrary, it indicates that the use of electric vehicles is relatively low. First, the results of location selection in the most ideal environment, such as the one with the smallest service radius, are obtained. Then, the service radius is further expanded for asymptotic optimization.

**Fig 3:**
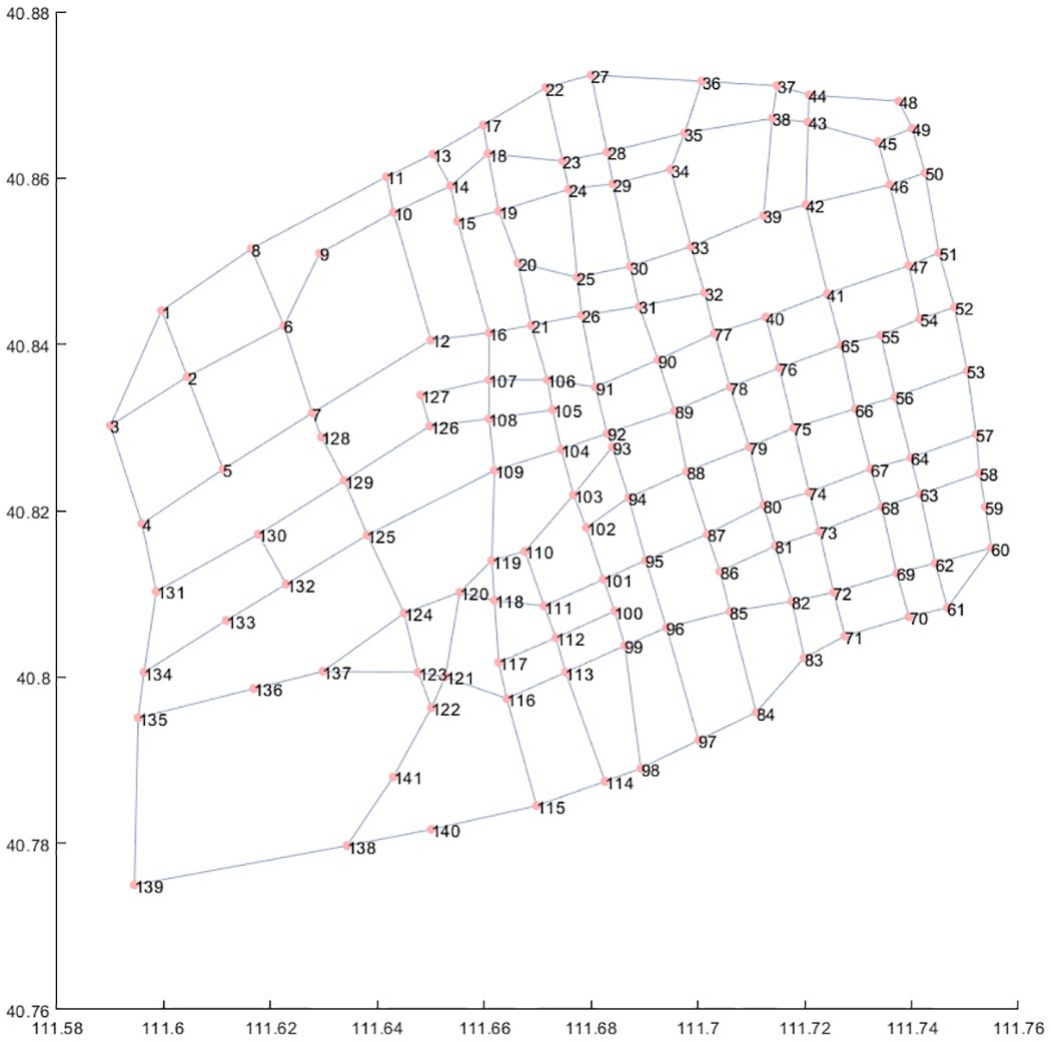
Transportation network of the city.

In practice, it is necessary to obtain basic data, candidate locations, and uncertain demand for urban transportation networks. These data can be used to validate the effectiveness of the LSCP model in the uncertain environment. The data of urban transportation networks can be collected from satellite maps or transportation departments. The candidate locations need to select based on the local characteristics of the city. A common option is to select locations near major road intersections. The associated demand can be predicted based on the permanent population near the location. The urban charging problem is strongly correlated with the number of electric vehicles, it can be assumed that charging at each location follows a Poisson distribution. This paper extracted the latitude and longitude information of 141 major transportation nodes in a Chinese city and stored this information in Appendix. Since intersections in urban are basically connected in a straight line, the actual distance between intersections can be calculated using latitude and longitude distances. Based on the actual distance between intersections, the Floyd algorithm can be used to calculate the shortest path between any two demand locations. The specific results are shown in Fig 3:

### Results of the experiment

In this paper, the set of service radii chosen contains 1 KM, 2 KM and 3 KM. The set can be used to describe different optimization results in different environments. There are two situations in which the asymptotic methods currently adopted are used. In the forward method, this paper can use the forward method from large to small service radius to optimize the location plan. The main idea is that the location results obtained in the case of a large service radius are used as part of the results for locations with a small service radius. Specifically, the location results obtained at a 3 KM service radius are directly used as the 2 KM location, and so on. In the contrary, the backward method uses the service radius from small to large to optimize the location result.

First, it is necessary to plan the location plan for the final stage with a service radius of 1 KM. Based on the plan, the selected node is used as candidate nodes under 2 KM service radius. Similarly, the 2 KM location result is used as candidate nodes for 3 KM service radius. With the promotion of electric vehicles, the demand for charging stations is gradually increasing. The demand of 1 KM service radius will be reached. In the future, the endurance of electric vehicles will increase, and the cost of home charging stations will shrink. They will lead to a decline in demand for communal charging stations. Eventually the demand will probably stabilize at a service radius of 2 KM. Therefore, when the result under maximum demand is given, timely adjustments can be made according to changes in the real situation. It can reduce the idleness of facilities and avoid the waste of resources. Both methods of asymptotic balance are important measures that can alleviate wasted resources and increased costs. The results are also suitable for promotion in practical applications. This paper is concerned with the dynamic planning problem. Therefore, it focuses on the balance and cost of the location plan in the middle and late stages of construction. In this paper, model 6 and model 9 are used to initialize the two asymptotic methods. Further planning using improved models for different situations to select more reasonable plans.

In the model, *ε* is the upper bound of the relaxation balance constrain level. It measures the extent to which the balance constraint is relaxed. As *ε* increases, the model balance constraint is gradually relaxed. And cost is a key component to consider. A reasonable location plan should ensure both the maximum balance and the minimum total cost. This paper establishes two evaluation indexes to analyze the balance and cost to find the most reasonable location plan.

First, this paper establishes the following index to measure the balance of workload:

$$E=\frac{\varepsilon /P}{W_{all}/P}=\frac{\varepsilon }{W_{all}}$$

The *E* is the percentage of average bias for each selected facility. The *ε/P* is the extent bias to each selected facility. And the *W*_*all*_/*P* is the average workload to each selected facility. As the *E* increases, the more bias is distributed to each selected facility, and the results are more poorly balanced. In addition, for simulation experiments, this paper sets the parameters *α* and *β* of the ideal workload to be both 0.5. Table 3 shows the different results obtained by selecting different initial *ε*.

**Table 3:** Balance values for different initial *ε* values.

Initial *ε*	Asymptotic method	Model	1 KM	2 KM	3 KM
*ε* = 0.1*W_***opt***_	Forward	Model 10	15.18%	2.14%	0.20%
		Model 11	75.90%	4.27%	0.17%
	Backward	Model 12	15.18%	2.14%	7.40%
		Model 13	15.18%	2.14%	7.47%
	\	LSCP	54.33%	45.60%	48.00%
*ε* = 0.2*W_***opt***_	Forward	Model 10	26.36%	6.05%	0.32%
		Model 11	123.60%	9.99%	0.32%
	Backward	Model 12	26.36%	6.05%	13.60%
		Model 13	26.36%	6.16%	1.52%
	\	LSCP	50.20%	50.20%	50.20%
*ε* = 0.3*W_***opt***_	Forward	Model 10	27.69%	6.66%	0.96%
		Model 11	71.28%	13.39%	0.96%
	Backward	Model 12	27.69%	6.66%	3.84%
		Model 13	27.69%	9.55%	1.44%
	\	LSCP	39.30%	19.95%	21.00%

From the Table 3 above, the value of bias *E* gradually increases with increasing *ε*. In addition, 3 KM is the initial construction stage of the forward method. As the service radius decreases, the number of facilities and the value of *E* are increasing. It indicates that the balance becomes worse. When the service radius is 1 KM, the balance of the results in the uncertain environment is more than 70%. It indicates the balance is very poor. In the contrary, 1 KM is the initial planning result of the backward method and the final stage of construction. In the backward method, the value of *E* has been decreasing and is less than 30% in all cases as the service radius increases. The balance is better. The 2 KM service radius as an intermediate stage common to both asymptotic methods. In the future, it is the final stabilization stage. By comparing the balance of the forward and backward methods at 2 KM, the latter is better in balance in both certain and uncertain environments.

The above comparison is the balance of location plan. However, cost is also the focus of this paper. It is the most reasonable optimization result to reach the balance optimum and the minimum of the cost at the same time. In this paper, the unit-weighted transportation cost *C*_*ij*_ is taken to be 2. The construction cost *C*^*’*^_*j*_ is influenced by factors such as the price of land in the area. In general, the closer to the center of the city, the higher the construction cost will be. This paper comprehensively analyzes the two asymptotic methods by means of cost improvement index. The index is constructed as shown below:

$$im=\frac{f_{c}-b_{c}}{f_{c}}$$

Where *im* is the percentage improvement of the backward method over the forward method. The *f*_*c*_ and *b*_*c*_ are the total costs of the forward and backward approaches, respectively. In case of *im*>0, it means that the cost of the backward method is lower than the forward method. In the contrary when *im*<0, it indicates that the total cost of the backward method is higher than the forward method. The total cost for different initial *ε* values is shown in Table 4.

**Table 4:** Total cost for different initial *ε* values.

Initial *ε*	Asymptotic method	Model	1 KM	2 KM	3 KM	Total cost
**\**	**\**	LSCP	1.46E+07	4.67E+06	2.52E+06	1.46E+07
*ε* = 0.1*W_***opt***_	**Forward**	Model 10	9.68E+06	3.55E+06	2.27E+06	1.55E+07
		Model 11	6.50E+06	7.70E+06	2.13E+06	1.63E+07
	**Backward**	Model 12	1.06E+07	1.91E+06	3.31E+06	1.59E+07
		Model 13	9.85E+06	2.39E+06	2.26E+06	1.45E+07
*ε* = 0.2*W_***opt***_	**Forward**	Model 10	6.61E+06	6.95E+06	2.63E+06	1.62E+07
		Model 11	7.89E+06	7.20E+06	2.55E+06	1.76E+07
	**Backward**	Model 12	1.11E+07	1.91E+06	2.56E+06	1.56E+07
		Model 13	9.02E+06	3.01E+06	1.89E+06	1.39E+07
*ε* = 0.3*W_***opt***_	**Forward**	Model 10	9.21E+06	5.09E+06	2.76E+06	1.71E+07
		Model 11	8.09E+06	7.51E+06	2.45E+06	1.81E+07
	**Backward**	Model 12	1.06E+07	2.48E+06	2.40E+06	1.55E+07
		Model 13	9.28E+06	2.74E+06	2.01E+06	1.40E+07

From Table 4 below, the total cost of the two asymptotic methods is less than that of the LSCP model. The forward method obtains the least cost plan under a small initial *ε* in both certain and uncertain environments. However, the total cost of the backward method is less volatile under different initial *ε*.

Overall, the total cost of the backward method is less than that of the forward method. To further analyze the reasons, the cost improvement index at each stage and in total is calculated. The cost improvement index for different initial *ε* values is shown in Table 5.

**Table 5:** Cost improvement index for different initial *ε* values.

Initial *ε*	Environment	1 KM	2 KM	3 KM	Total cost
*ε* = 0.1*W_***opt***_	Certain	-0.10	0.46	-0.46	-0.02
	Uncertain	-0.52	0.69	-0.06	0.11
*ε* = 0.2*W_***opt***_	Certain	-0.68	0.73	0.03	0.04
	Uncertain	-0.14	0.58	0.26	0.21
*ε* = 0.3*W_***opt***_	Certain	-0.15	0.51	0.13	0.09
	Uncertain	-0.15	0.64	0.18	0.22

From Table 5 above, there is a high probability that the total cost of the backward method is lower than the forward method for different initial *ε*. However, the total cost of the backward method is higher in the certain environment and *ε* = 0.3*W_*opt*_. In the certain and uncertain environments, the forward and backward methods are more obvious in cost. When the demand is high, the cost of the forward method is smaller. Conversely, when demand is low, the cost of the backward method is lower.

When both the balance and the cost improvement index are analyzed together, the two methods have their own advantages. First, the forward method has a better balance and lower cost at 3 KM service radius and *ε* = 0.1*W_*opt*_. Second, as the demand increases, the backward method is better in cost and balance at a service radius of 2 KM. Finally, at the 1 KM service radius stage, the cost of the forward method is lower. However, the balance is worse than the backward method. It can increase the asymmetry. In general, in practical management, choosing different location plans can effectively save costs and avoid wasting resources.

**Fig 4:**
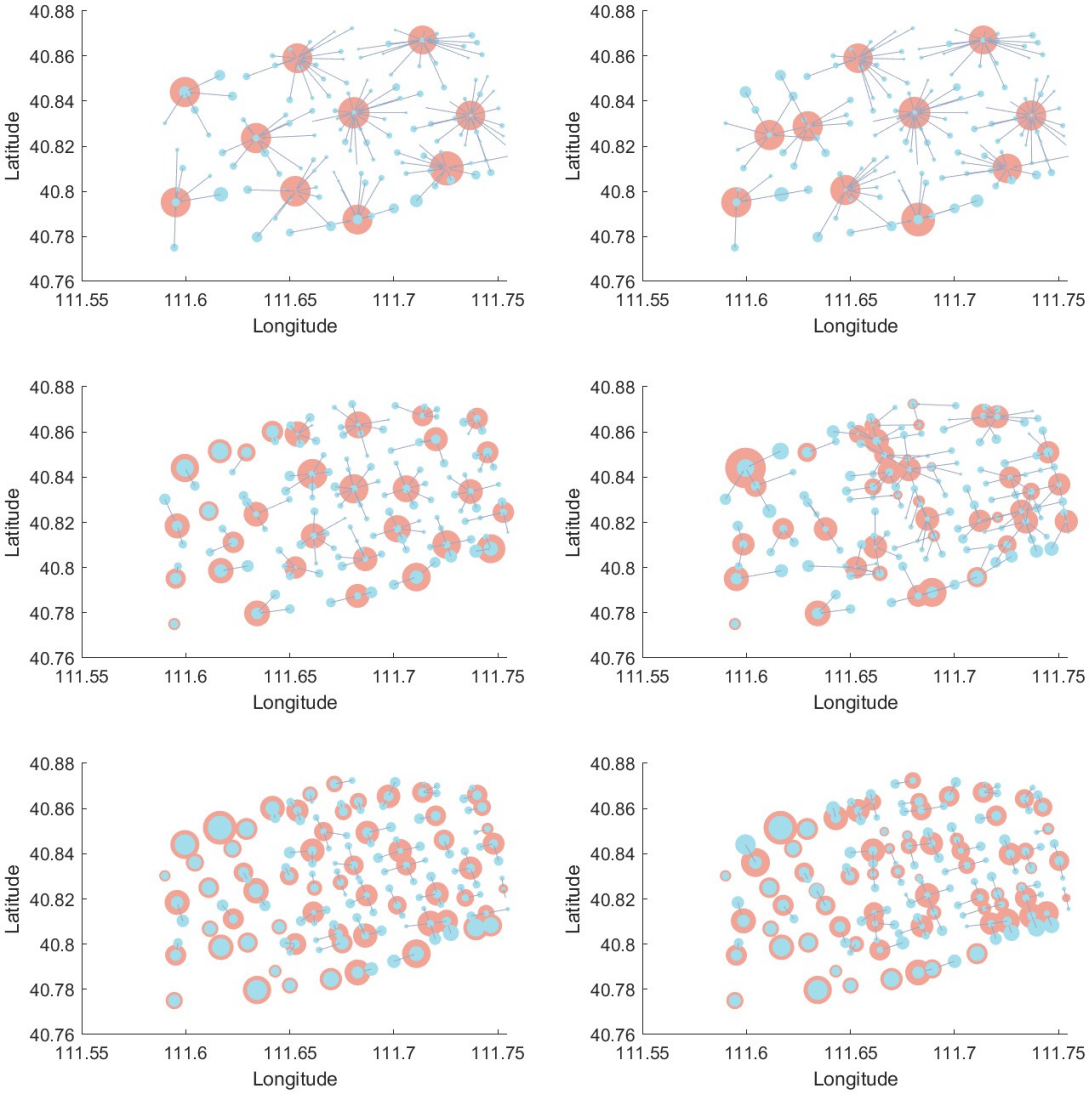
Visualization of the forward method.

**Fig 5:**
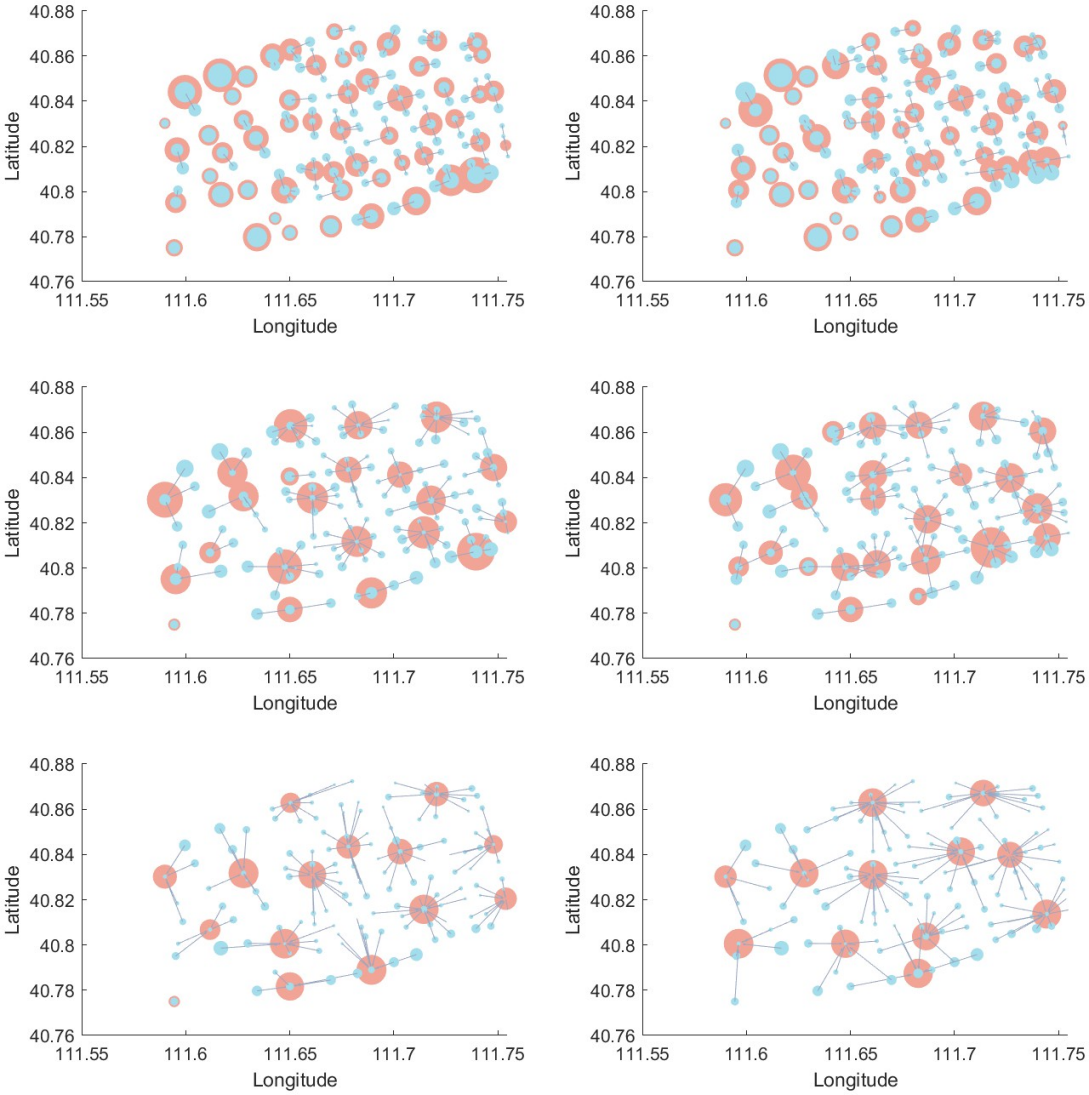
Visualization of the backward method.

In this paper, the CPLEX solver is used to solve the problem and visualized by MATLAB. The location results of the two asymptotic methods at different service radius for initial *ε* = 0.2*W_*opt*_ are shown in Figs 4 and 5, respectively. The blue and red dots are the demand and facility nodes, respectively. The size of the blue dot is the demand for that node. The size of the red dot is the workload of the facility. In Fig 4, the left and right sides represent the results of Model 10 and Model 11, respectively. In Fig 5, the left and right sides show the results for Model 12 and Model 13, respectively.

## Conclusion

This paper introduces the asymptotic BLSCP model in uncertain and asymmetric environments in combination with the traditional LSCP model. The models consider not only the problem of increasing demand and the uncertainty characteristic of stabilization tendency, but also the asymmetry of workloads at different locations. Through the theory of goal programming, the dual objectives of balancing the workload of the facilities and minimizing the costs have been achieved. In addition, in terms of the number of locations, the optimal number of locations and plans for different stages have been obtained by considering the cost and coverage factors. In determining the ideal workload for each facility, a minimum upper bound and a maximum lower bound for the workload at the specified number of locations were obtained by introducing appropriate models. Based on the upper and lower bounds, a better ideal workload for the facility was designed. Finally, the asymptotic location problem with increasing demand was solved using the backward and forward methods. In this paper, the charging station location problem in a city is simulated as an example. The results show that the backward method performs better in both balance and cost as demand increases. Although the forward method performs better in cost at the final stage, the total cost has no advantage. And the forward method has a worse balance in the final stage under the uncertain environment.

Based on the traditional LSCP model, this paper further considers uncertainty, asymmetry, and increased demand in the proposed models. The goal of more realistic location requirements was achieved. The potential minimum and maximum workloads for different candidate locations were obtained through simulation. Based on the simulation results, the goal of balancing the facility workload is achieved. In addition, the multi-objective consideration of location cost, transportation cost, and balance of facility workload in this paper further expands the potential generalization value. In the future, the exploration of fast solution algorithms for models in large-scale backgrounds, more diverse theoretical generalizations of models, and realistic applied research are of great value.

## Supporting information

S1 Appendix 141 candidate locations and the relevant data. (DOCX)
