# Supplementary material for: Asymptotic BLSCP models in uncertain and asymmetric environments—Take the charging station location problem
Source: PLoS One. 2025 Jan 24;20(1):e0305669. doi: 10.1371/journal.pone.0305669 (PMC11760035; doi:10.1371/journal.pone.0305669)
Supplement: S1 Appendix — (DOCX) [file pone.0305669.s001.docx]

## Appendix

**Table A.1**

141 candidate locations and the relevant data.

| Number | Longitude | Latitude | Workload_av | Link point |
| --- | --- | --- | --- | --- |
| 1 | 111.5996 | 40.84405 | 11.868 | 2,3,8 |
| 2 | 111.6044 | 40.83611 | 11.67 | 1,3,5,6 |
| 3 | 111.59 | 40.83021 | 5.706 | 1,2,4 |
| 4 | 111.5959 | 40.81842 | 9.006 | 3,5,131 |
| 5 | 111.6111 | 40.82497 | 12.48 | 2,4,7 |
| 6 | 111.6225 | 40.84222 | 10.82 | 2,7,8,9 |
| 7 | 111.6278 | 40.83174 | 9.441 | 5,6,12,128 |
| 8 | 111.6164 | 40.8515 | 10.17 | 1,6,11 |
| 9 | 111.6292 | 40.85095 | 15.64 | 6,10 |
| 10 | 111.643 | 40.85586 | 8.1 | 9,11,12,14 |
| 11 | 111.6417 | 40.86015 | 2.944 | 8,10,13 |
| 12 | 111.65 | 40.8405 | 10.11 | 7,10,16 |
| 13 | 111.6504 | 40.86287 | 2.828 | 11,14,17 |
| 14 | 111.6537 | 40.85906 | 2.81 | 10,13,15,18 |
| 15 | 111.655 | 40.8548 | 5.78 | 14,16,19 |
| 16 | 111.6609 | 40.84137 | 5.18 | 12,15,21,107 |
| 17 | 111.6598 | 40.86637 | 1.533 | 13,18,22 |
| 18 | 111.6607 | 40.86293 | 3.68 | 14,17,19,23 |
| 19 | 111.6627 | 40.85603 | 4.64 | 15,18,20,24 |
| 20 | 111.6663 | 40.84981 | 5.23 | 19,21,25 |
| 21 | 111.6688 | 40.84227 | 4.2 | 16,20,26,106 |
| 22 | 111.6715 | 40.87087 | 4.0185 | 17,23,27 |
| 23 | 111.6747 | 40.86203 | 4.36 | 18,22,24,28 |
| 24 | 111.6758 | 40.85865 | 4.49 | 19,23,25,29 |
| 25 | 111.6774 | 40.84806 | 5.04 | 20,24,26,30 |
| 26 | 111.6782 | 40.84348 | 4.07 | 21,25,31,91 |
| 27 | 111.68 | 40.87239 | 3.42 | 22,28,36 |
| 28 | 111.683 | 40.86312 | 4.72 | 23,27,29,35 |
| 29 | 111.6841 | 40.8593 | 4.38 | 24,28,30,34 |
| 30 | 111.6873 | 40.84937 | 5.1 | 25,29,31,33 |
| 31 | 111.689 | 40.84457 | 4.66 | 26,30,32,90 |
| 32 | 111.7012 | 40.8462 | 4.79 | 31,33,77 |
| 33 | 111.6987 | 40.85177 | 5.79 | 30,32,34,39 |
| 34 | 111.6949 | 40.86105 | 5.56 | 29,33,35 |
| 35 | 111.6975 | 40.86541 | 6.49 | 28,34,36,38 |
| 36 | 111.7007 | 40.87163 | 3.63 | 27,35,37 |
| 37 | 111.7148 | 40.87109 | 1.786 | 36,38,44 |
| 38 | 111.7139 | 40.86716 | 5.58 | 35,37,39,43 |
| 39 | 111.7123 | 40.85548 | 8.22 | 33,38,42 |
| 40 | 111.7127 | 40.84326 | 6.16 | 41,76,77 |
| 41 | 111.7242 | 40.84609 | 7.29 | 40,42,47,65 |
| 42 | 111.7202 | 40.85679 | 7.12 | 39,41,43,46 |
| 43 | 111.7206 | 40.86672 | 4.46 | 38,42,44,45 |
| 44 | 111.7208 | 40.86999 | 1.614 | 37,43,48, |
| 45 | 111.7337 | 40.86432 | 5.29 | 43,46,49, |
| 46 | 111.7359 | 40.85919 | 5.56 | 42,45,47,50 |
| 47 | 111.7395 | 40.84948 | 4.94 | 41,46,51,54 |
| 48 | 111.7376 | 40.86923 | 1.54 | 44,49 |
| 49 | 111.7401 | 40.86596 | 1.268 | 45,48,50 |
| 50 | 111.7425 | 40.86061 | 2.667 | 46,49,51 |
| 51 | 111.7451 | 40.85101 | 2.079 | 47,50,52 |
| 52 | 111.748 | 40.84446 | 2.044 | 51,53,54 |
| 53 | 111.7504 | 40.83682 | 4.424 | 52,56,57 |
| 54 | 111.7415 | 40.84304 | 3.8 | 47,52,55 |
| 55 | 111.7342 | 40.84107 | 4.26 | 54,56,65 |
| 56 | 111.7369 | 40.83365 | 5.06 | 53,55,64,66 |
| 57 | 111.7521 | 40.82917 | 3.222 | 53,58,64 |
| 58 | 111.7527 | 40.82448 | 2.061 | 57,59,63 |
| 59 | 111.7538 | 40.82044 | 2.014 | 58,60 |
| 60 | 111.7549 | 40.81553 | 1.568 | 59,61,62 |
| 61 | 111.7467 | 40.80832 | 2.113 | 60,62,70 |
| 62 | 111.7444 | 40.81367 | 3.93 | 60,61,63,69 |
| 63 | 111.7416 | 40.82197 | 4.3 | 58,62,64,68 |
| 64 | 111.7398 | 40.82634 | 4.16 | 56,57,63,67 |
| 65 | 111.7268 | 40.83987 | 4.14 | 41,55,66,76 |
| 66 | 111.7294 | 40.83223 | 5.18 | 56,65,67,75 |
| 67 | 111.7323 | 40.82503 | 3.96 | 64,66,68,74 |
| 68 | 111.7344 | 40.82044 | 3.48 | 63,67,69,73 |
| 69 | 111.7372 | 40.81247 | 4.3 | 62,68,70,72 |
| 70 | 111.7395 | 40.80723 | 1.984 | 61,69,70,71 |
| 71 | 111.7275 | 40.80493 | 3.576 | 70,72,83 |
| 72 | 111.7254 | 40.81017 | 4.18 | 69,71,73,82 |
| 73 | 111.7227 | 40.81749 | 4.27 | 68,72,74,81 |
| 74 | 111.7208 | 40.8222 | 4.01 | 67,73,75,80 |
| 75 | 111.7179 | 40.82994 | 5.02 | 66,74,76,79 |
| 76 | 111.7152 | 40.83714 | 4.29 | 40,65,75,78 |
| 77 | 111.7031 | 40.84129 | 4.16 | 32,40,78,90 |
| 78 | 111.706 | 40.83485 | 5.05 | 76,77,79,89 |
| 79 | 111.7096 | 40.82765 | 4.93 | 75,78,80,88 |
| 80 | 111.7123 | 40.82066 | 4.2 | 74,79,81,87 |
| 81 | 111.7145 | 40.81576 | 3.82 | 73,80,82,86 |
| 82 | 111.7176 | 40.80908 | 4.72 | 72,81,83,85 |
| 83 | 111.7199 | 40.80231 | 4.956 | 71,82,84 |
| 84 | 111.7109 | 40.79576 | 5.154 | 83,85,97 |
| 85 | 111.706 | 40.80788 | 6.78 | 82,84,86,96 |
| 86 | 111.7041 | 40.81269 | 3.62 | 81,85,87 |
| 87 | 111.7017 | 40.81716 | 5.01 | 80,86,88,95 |
| 88 | 111.6979 | 40.8247 | 5.76 | 79,87,89,94 |
| 89 | 111.6956 | 40.83201 | 5.56 | 78,88,90,92 |
| 90 | 111.6925 | 40.83813 | 4.94 | 31,77,89,91 |
| 91 | 111.6808 | 40.83485 | 4.54 | 26,90,92,106 |
| 92 | 111.683 | 40.82928 | 2.68 | 89,91,93,104 |
| 93 | 111.684 | 40.82767 | 2.9 | 92,94,103 |
| 94 | 111.687 | 40.82156 | 4.16 | 88,93,95,102 |
| 95 | 111.6902 | 40.81402 | 4.64 | 87,94,96,101 |
| 96 | 111.6941 | 40.80594 | 6.98 | 85,95,97,99 |
| 97 | 111.7001 | 40.7924 | 5.277 | 84,96,98 |
| 98 | 111.6893 | 40.78901 | 5.08 | 97,99,114 |
| 99 | 111.6863 | 40.80376 | 5.77 | 96,98,100,113 |
| 100 | 111.6844 | 40.80791 | 2.83 | 99,101,112 |
| 101 | 111.6823 | 40.81173 | 3.63 | 95,100,102,111 |
| 102 | 111.6791 | 40.81796 | 3.94 | 94,101,103 |
| 103 | 111.6767 | 40.82189 | 3.28 | 93,102,104,110 |
| 104 | 111.6744 | 40.82735 | 3.78 | 92,103,105,109 |
| 105 | 111.6728 | 40.83212 | 2.88 | 104,106,108 |
| 106 | 111.6718 | 40.83572 | 3.51 | 21,91,105,107 |
| 107 | 111.6609 | 40.83572 | 3.64 | 16,106,108,127 |
| 108 | 111.6609 | 40.83103 | 4.09 | 105,107,109,126 |
| 109 | 111.6619 | 40.82482 | 7.24 | 104,108,119,125 |
| 110 | 111.6676 | 40.8151 | 5.16 | 103,111,119 |
| 111 | 111.6712 | 40.80854 | 3.99 | 101,110,112,118 |
| 112 | 111.6734 | 40.80471 | 3.05 | 100,111,113,117 |
| 113 | 111.6752 | 40.80056 | 6.99 | 99,112,114,116 |
| 114 | 111.6826 | 40.78745 | 4.844 | 98,113,115 |
| 115 | 111.6698 | 40.78451 | 8.315 | 114,116,140 |
| 116 | 111.6642 | 40.7974 | 7.78 | 113,115,117,121 |
| 117 | 111.6627 | 40.80176 | 4.01 | 112,116,118 |
| 118 | 111.6619 | 40.80919 | 3.05 | 111,117,119,120 |
| 119 | 111.6614 | 40.814 | 3.85 | 109,110,118,120 |
| 120 | 111.6554 | 40.81017 | 7.12 | 118,119,121,124 |
| 121 | 111.6527 | 40.80002 | 4.27 | 116,120,122,123 |
| 122 | 111.6501 | 40.7963 | 5.27 | 121,123,141 |
| 123 | 111.6476 | 40.80056 | 5.26 | 121,122,124,137 |
| 124 | 111.645 | 40.80766 | 7.92 | 120,123,125,137 |
| 125 | 111.6381 | 40.81705 | 10.64 | 109,124,129,132 |
| 126 | 111.6498 | 40.83016 | 8.54 | 108,127,129 |
| 127 | 111.6481 | 40.83387 | 6.66 | 107,126 |
| 128 | 111.6295 | 40.82885 | 5.73 | 7,129 |
| 129 | 111.6338 | 40.82361 | 7.67 | 125,126,128,130 |
| 130 | 111.6177 | 40.81716 | 8.32 | 129,131,132 |
| 131 | 111.5986 | 40.81028 | 6.405 | 4,130,134 |
| 132 | 111.6229 | 40.81116 | 9.21 | 125,130,133 |
| 133 | 111.6117 | 40.80679 | 9.92 | 132,134 |
| 134 | 111.5963 | 40.80056 | 6.248 | 131,133,135 |
| 135 | 111.5952 | 40.7951 | 10.728 | 134,136,139 |
| 136 | 111.6169 | 40.7986 | 20.58 | 135,137 |
| 137 | 111.6298 | 40.80067 | 13.81 | 123,124,136 |
| 138 | 111.6343 | 40.7797 | 16.947 | 139,140,141 |
| 139 | 111.5945 | 40.775 | 32.087 | 135,138 |
| 140 | 111.6501 | 40.78166 | 7.758 | 115,138 |
| 141 | 111.643 | 40.788 | 9.35 | 122,138 |
